# Nutritional Risk in Patients Prepared for Surgical Treatment: A Cross-Sectional NRS 2002 Study

**DOI:** 10.3390/jcm15197377

**Published:** 2026-09-23

**Authors:** Marta Łuczyk, Robert Jan Łuczyk, Dorota Weber, Kamil Sikora, Karolina Weronika Tutak, Anna Charuta

**Affiliations:** 1 Long-Term Care Nursing Department, Chair of Preventive Nursing, Faculty of Health Sciences, Medical University of Lublin, 20-093 Lublin, Poland; 2 Institute of Health Sciences, Faculty of Medical and Health Sciences, University of Siedlce, 08-110 Siedlce, Poland; robert.luczyk@uws.edu.pl (R.J.Ł.); anna.charuta@uws.edu.pl (A.C.); 3 Faculty of Health Sciences, Lublin University of Technology, WSEI in Lublin, 20-209 Lublin, Poland; dorota.weber@wsei.pl; 4 Department of Internal Medicine and Internal Nursing, Chair of Preventive Nursing, Faculty of Health Sciences, Medical University of Lublin, 20-093 Lublin, Poland; kamil.sikora@umlub.edu.pl

**Keywords:** nutritional risk, Nutritional Risk Screening 2002, preoperative care, surgical patients, oncological surgery, ERAS, cross-sectional study

## Abstract

**Background/Objectives**: Preoperative nutritional status is a recognised determinant of postoperative outcomes, influencing wound healing, complication rates, and length of hospital stay. This study assessed nutritional risk among patients prepared for surgical treatment using the Nutritional Risk Screening 2002 (NRS 2002) tool and examined its association with sociodemographic characteristics, gastrointestinal symptoms, comorbidities, and malignancy diagnosis. **Methods**: A cross-sectional survey was conducted among 94 patients hospitalized on general/minimally invasive surgery and oncological surgery wards of a military clinical hospital in Lublin, Poland, between January and March 2025, using the NRS 2002 tool alongside a structured sociodemographic and clinical questionnaire. Normality was assessed using the Shapiro–Wilk test. Group comparisons used the Mann–Whitney U test for two groups and the Kruskal–Wallis H test for three or more groups, with post hoc pairwise comparisons where indicated; associations with age and treatment duration were assessed using Spearman’s rank correlation; and the association between malignancy diagnosis and nutritional risk category was assessed using the chi-square test with Cramér’s V. Analyses were performed in R 4.2.2/RStudio, with α set at 0.05. **Results**: Most patients (76.6%, *n* = 72; 95% confidence interval [CI]: 67.1–84.0%) showed no increased nutritional risk on the final NRS 2002 classification, while 23.4% (*n* = 22; 95% CI: 16.0–32.9%) had an indication for nutritional treatment. Nutritional risk score increased significantly with age (Spearman ρ = 0.633, *p* < 0.001), educational level (Kruskal–Wallis H(3) = 19.29, *p* < 0.001), marital status (H(3) = 17.70, *p* = 0.001), employment status (H(2) = 21.92, *p* < 0.001), and ward length of stay (H(3) = 36.18, *p* < 0.001). Nausea, vomiting, diarrhoea, and appetite loss were each associated with higher scores (all *p* ≤ 0.002), whereas heartburn was not. Hypertension, atherosclerosis, and chronic obstructive pulmonary disease were associated with elevated scores (*p* ≤ 0.045). The proportion meeting the nutritional-treatment threshold was numerically higher among patients with malignancy than among those without malignancy (32.6% vs. 15.7%), but this difference did not reach statistical significance (Pearson χ^2^(1) = 3.70, *p* = 0.054, Cramér’s V = 0.199). Analyses of admission mode and total illness duration were not statistically significant (*p* = 0.828 and *p* = 0.452, respectively). **Conclusions**: While most patients prepared for surgery had no increased nutritional risk according to NRS 2002, nearly one in four met the tool’s threshold for nutritional treatment. In exploratory unadjusted analyses, higher scores were observed among older, retired, widowed, and less-educated patients and among those reporting selected gastrointestinal symptoms or specific comorbidities. Patients with malignancy more often met the threshold for nutritional treatment, although this difference was not statistically significant in the present sample. These findings support systematic preoperative nutritional screening followed by further assessment of patients who screen positive, consistent with Enhanced Recovery After Surgery (ERAS) principles.

## 1. Introduction

Nutritional status has long occupied an ambiguous position in perioperative care: it is intuitively important to clinicians, yet historically under-assessed relative to more immediately visible surgical risk factors. This has changed substantially over the past two decades, as accumulating evidence has linked preoperative malnutrition to impaired wound healing, higher rates of surgical site infection and other postoperative complications, longer hospital stays, and increased treatment costs [1,2]. The 2025 update of the ESPEN guideline on clinical nutrition in surgery reinforces that nutritional risk should be identified early and treated as an integral component of perioperative care rather than as an ancillary concern [3]. The proportion of surgical patients affected is far from marginal: malnutrition or its risk has been documented in 30–50% of chronically ill patients at hospital admission generally, rising to 40–90% among oncological patients specifically, and, once present, has been shown to worsen further in around 70% of already-malnourished patients within just 14 days of admission if unaddressed [4]. Contemporary guidance for polymorbid inpatients likewise recommends systematic screening followed by individualized nutritional support when risk is detected [5].

Several validated instruments exist to screen for this risk at the bedside, including the Nutritional Risk Screening 2002 (NRS 2002), the Subjective Global Assessment (SGA), and the Mini Nutritional Assessment (MNA) in older populations specifically [6,7]. A recent network meta-analysis of preoperative screening instruments confirmed that no single brief tool is sufficient as a stand-alone diagnostic assessment and that patients screening positive should undergo more comprehensive nutritional evaluation [8]. Similarly, a 2024 systematic review and meta-analysis demonstrated meaningful differences in sensitivity and specificity among tools used in hospitalized adults, supporting the use of validated screening pathways rather than unstructured clinical judgement [9]. NRS 2002 in particular was developed with a hospitalized, largely surgical population in mind and combines a brief four-item screen with, for patients screening positive, a fuller assessment incorporating both the severity of nutritional impairment and the increased nutrient requirements associated with disease severity itself, making it well suited to preoperative use [10]. Beyond wound healing and infection risk, the health consequences of unaddressed malnutrition extend to impaired immune function, muscle wasting, and, in severe cases, increased mortality [11,12], while a range of complementary assessment methods—anthropometric, biochemical, and clinical—have been described specifically for oncological populations, reflecting the particular complexity of nutritional evaluation in patients undergoing cancer treatment [13].

The perioperative period presents particular nutritional challenges. Prolonged preoperative fasting, once routine practice extending many hours beyond what current evidence supports, has been progressively shortened under Enhanced Recovery After Surgery (ERAS) protocols, which explicitly incorporate nutritional optimisation—including reduced fasting duration and, where appropriate, preoperative carbohydrate loading—as a core element of reducing postoperative complications and accelerating recovery [14,15]. Current perioperative nutrition reviews and the updated ESPEN surgical guideline emphasize early oral feeding, avoidance of unnecessary fasting, and timely nutritional therapy in patients identified as being at risk [3,16,17]. ERAS-concordant nutritional care has demonstrated benefit across multiple surgical specialties, including colorectal and oesophageal cancer resection [18,19], yet its effective implementation presupposes that patients at elevated nutritional risk are first correctly identified, which in routine practice depends on consistent application of a validated screening tool such as NRS 2002 rather than on clinical impression alone.

The present study aimed to assess nutritional risk, using the NRS 2002 tool, among patients hospitalized in preparation for surgical treatment on general, minimally invasive, and oncological surgery wards, and to identify the sociodemographic, clinical, and disease-related factors associated with elevated risk in this population, including the contribution of malignancy diagnosis.

## 2. Materials and Methods

### 2.1. Study Design and Setting

This cross-sectional survey was conducted between January and March 2025 among patients hospitalized on the general and minimally invasive surgery ward and the oncological surgery ward of the 1st Military Clinical Hospital with Polyclinic SPZOZ in Lublin, Poland.

### 2.2. Participants

Participants were recruited using convenience sampling from available patients hospitalized in preparation for surgical treatment on the two study wards during the recruitment period [20,21]. Eligibility required age ≥ 18 years, hospitalization on one of these wards, preparation for surgical treatment, and the ability to understand and answer the questionnaire. Patients who were unable to provide responses were not included. A total of 94 completed questionnaires were obtained; the number of patients who declined participation was not recorded.

### 2.3. Instruments

Nutritional risk was screened using the Nutritional Risk Screening 2002 (NRS 2002) questionnaire. NRS 2002 is a screening instrument: a positive result identifies a patient who requires further nutritional assessment and may indicate nutritional treatment; it does not by itself establish a definitive diagnosis of malnutrition. The instrument’s initial screening component comprises four yes/no items: body mass index below 20.5 kg/m^2^; unintentional weight loss within the preceding three months; reduced food intake over the preceding week; and presence of severe acute illness. Patients answering “yes” to at least one screening item proceed to the full assessment, which separately scores (0–3 points each) the severity of nutritional impairment and the disease-related increase in nutrient requirements; one additional point is added to the total score for patients aged over 70 years. Participants with no positive response at the initial screening stage were classified as not requiring nutritional treatment on the basis of the initial screen. A separate flow count of patients proceeding to the full assessment was not retained in the study records. A total score of 3 or more points was treated as an indication for nutritional treatment and further assessment, whereas a score below 3 indicated the need for conservative management with re-screening after one week. The NRS 2002 questionnaire was supplemented by a 28-item structured questionnaire of original design, collecting sociodemographic data (age, sex, education, marital status, place of residence, employment and housing situation), self-rated health and physical capacity, reason for hospitalization, gastrointestinal symptoms, comorbidities, oncological treatment history, and admission characteristics (mode of admission, illness duration, and ward length of stay at the time of questionnaire completion).

### 2.4. Statistical Analysis

Statistical analyses were performed using the R programming language, version 4.2.2, within the RStudio environment, version 2022.12.0. A significance threshold of α = 0.05 was applied throughout. Descriptive statistics for continuous variables (age, weight, height, treatment duration, and NRS 2002 component scores) are reported as means, standard deviations, medians, skewness, and kurtosis; categorical and remaining demographic/clinical variables are reported as frequencies and percentages. The Shapiro–Wilk test was used to assess normality. For variables not meeting normality assumptions, group comparisons involving two categories used the Mann–Whitney U test (with epsilon-squared, ε^2^, as the effect size), whereas comparisons involving three or more categories used the Kruskal–Wallis H test (also reported with ε^2^), followed by post hoc pairwise comparisons where the omnibus test was significant. Associations between nutritional risk score and age or treatment duration were assessed using Spearman’s rank correlation coefficient (ρ). The association between nutritional risk category and malignancy diagnosis was assessed using the chi-square test with Cramér’s V as the effect size. The malignancy comparison also reports an unadjusted risk ratio with a 95% confidence interval calculated from the 2 × 2 table. Ninety-five percent confidence intervals for proportions are reported for the final NRS 2002 risk classification. Given the modest sample size and sparse subgroups, analyses were restricted to unadjusted bivariate comparisons; no multivariable model was fitted. No formal correction for multiple comparisons was applied, so inferential findings were interpreted as exploratory.

### 2.5. Ethical Considerations

Participation in the anonymous survey was voluntary. Patients were informed of the study’s anonymous nature and academic purpose before completing the questionnaire. No personally identifiable information was collected, and the study involved no experimental intervention or alteration of patient care. In accordance with applicable institutional and national regulations, formal approval from an institutional bioethics committee was not required for this anonymous questionnaire study.

## 3. Results

### 3.1. Sample Characteristics

The sample comprised 94 patients, slightly more often male (51.1%, *n* = 48) than female (48.9%, *n* = 46). The largest age band was 71–80 years (31.9%), followed by 61–70 years (18.1%), 51–60 years (16.0%), over 80 years (10.6%), 18–29 and 41–50 years (8.5% each), and 30–40 years (6.4%, the smallest group). Educational attainment was most often vocational (38.3%), followed by secondary (28.7%), higher (24.5%), and primary (8.5%). Place of residence was evenly split between urban and rural areas (50.0% each). Most patients were married (55.3%), with smaller proportions single (19.1%), widowed (18.1%), or divorced (7.4%); half were retired (50.0%), 45.7% were employed, and 4.3% were unemployed. Most patients lived with a spouse and/or children (55.3%), a smaller proportion with other family members (23.4%), and the remainder alone (21.3%). Self-rated general health was most often good (42.6%) or poor (39.4%), with very good ratings being the least common (18.1%; median = 2.0 on a 1–3 scale). Self-rated physical capacity was most often rated poor (62.8%), with sufficient (19.1%), good (12.8%), and insufficient (5.3%) ratings being less common (median = 1.0 on a 1–4 scale). Characteristics of the sample is presented on Table 1.

Most patients reported eating regularly (74.5%) and consuming three (41.5%) or four or more (38.3%) meals daily, with 20.2% consuming only one to two meals daily. The most common reason for hospitalization was a gastrointestinal disorder (56.4%), followed by thyroid disease (9.6%), abdominal or inguinal hernia (8.5%), appendectomy and cholecystectomy (6.4% each), breast tumour excision (4.3%), and smaller proportions for lower-limb varicose veins, metastatic lymph node removal, and skin lesion excision. Reasons for hospital admission of patients are presented on Table 2.

### 3.2. NRS 2002 Screening and Risk Classification

On the initial four-item NRS 2002 screen, 31.9% of patients (*n* = 30) had a BMI below 20.5 kg/m^2^, 41.5% (*n* = 39) reported unintentional weight loss within the preceding three months, and 27.7% (*n* = 26) reported reduced food intake over the preceding week; no patient was classified as severely acutely ill (0%, *n* = 0).

Among participants with recorded full-assessment scores, the mean nutritional impairment severity score was 0.44 (SD = 0.58, median 0.00, range 0–2), and the mean disease-severity/increased-requirement score was 0.51 (SD = 0.71, median 0.00, range 0–2). Both distributions departed significantly from normality (Shapiro–Wilk W = 0.683 and W = 0.690, respectively, both *p* < 0.001) and were right-skewed, reflecting a predominance of low (zero) scores. The combined total nutritional risk score (summing both components, with the age adjustment where applicable) had a mean of 1.34 (SD = 1.43, median 1.00, range 0–4) and was also significantly non-normal (W = 0.805, *p* < 0.001).

On the final NRS 2002 classification, 76.6% of patients (*n* = 72; 95% Wilson CI: 67.1–84.0%) showed no increased nutritional risk, while 23.4% (*n* = 22; 95% Wilson CI: 16.0–32.9%) had a formal indication for nutritional treatment (Table 3).

### 3.3. Nutritional Risk by Sociodemographic Characteristics

Nutritional risk score did not differ significantly by sex (Mann–Whitney U = 1071.0, *p* = 0.806, ε^2^ = 0.010; men M = 1.40 ± 1.50, women M = 1.28 ± 1.36) or place of residence (U = 915.0, *p* = 0.152, ε^2^ = 0.011; rural M = 1.53 ± 1.40, urban M = 1.15 ± 1.44). In contrast, educational level showed a strong, significant unadjusted association (H(3) = 19.29, *p* < 0.001, ε^2^ = 0.181): risk was highest among patients with primary education (M = 2.75 ± 0.89) and lowest among those with secondary or higher education (M = 0.78 ± 1.19 and M = 0.78 ± 1.13, respectively), with vocational education intermediate (M = 1.81 ± 1.51); post hoc comparisons confirmed significant differences between primary and secondary education (*p* = 0.002), primary and higher education (*p* = 0.002), secondary and vocational education (*p* = 0.010), and higher and vocational education (*p* = 0.011).

Marital status was similarly associated with nutritional risk (H(3) = 17.70, *p* = 0.001, ε^2^ = 0.163): widowed patients showed the highest risk (M = 2.00 ± 1.41), followed by married patients (M = 1.58 ± 1.42), single patients (M = 0.56 ± 1.10), and divorced patients, who showed the lowest risk (M = 0.00 ± 0.00); post hoc comparisons confirmed significant pairwise differences (all *p* ≤ 0.010). Employment status also showed an unadjusted association (H(2) = 21.92, *p* < 0.001, ε^2^ = 0.219), with retirees showing higher risk (M = 2.00 ± 1.34) than employed patients (M = 0.74 ± 1.24) or unemployed patients (M = 0.00 ± 0.00, though this subgroup was very small, *n* = 4). Housing situation was not significantly associated with nutritional risk (H(2) = 4.50, *p* = 0.106, ε^2^ = 0.027). Age correlated strongly and significantly with nutritional risk score (Spearman ρ = 0.633, *p* < 0.001). Because age was not controlled for in these bivariate comparisons, the sociodemographic associations should not be interpreted as independent effects. Associations between sociodemographic and admission variables are presented on Table 4.

### 3.4. Nutritional Risk by Admission Characteristics and Gastrointestinal Symptoms

Admission mode (urgent versus planned) was not significantly associated with nutritional risk score (Mann–Whitney U = 1018.0, *p* = 0.828, ε^2^ = 0.001), despite a numerically higher mean among urgently admitted patients (M = 1.41 ± 1.56) than those admitted on a planned basis (M = 1.31 ± 1.36). Total illness duration (mean 3.62 months, SD = 3.10, median 3.00, range 1–18, significantly right-skewed and non-normal per Shapiro–Wilk, W = 0.796, *p* < 0.001) likewise showed no significant correlation with nutritional risk (Spearman ρ = 0.079, *p* = 0.452). The duration of the current ward stay recorded at the time of questionnaire completion was strongly associated with risk (H(3) = 36.18, *p* < 0.001, ε^2^ = 0.369): patients who had been hospitalized for 8–14 days showed the highest risk (M = 2.60 ± 0.97), compared with lower risk among those staying under 3 days (M = 0.00 ± 0.00), 3–7 days (M = 0.77 ± 1.23), or over 14 days (M = 1.00 ± 1.41, though this longest-stay subgroup was very small, *n* = 2); post hoc comparisons confirmed significant differences between the under-3-day and 8–14-day groups (*p* = 0.031) and between the 3–7-day and 8–14-day groups (*p* < 0.001). Because duration was recorded cross-sectionally, the direction and clinical meaning of this association cannot be established.

Among gastrointestinal symptoms, diarrhoea (Mann–Whitney U = 315.0, *p* = 0.002, ε^2^ = 0.100), nausea (U = 341.5, *p* < 0.001, ε^2^ = 0.182), vomiting (U = 316.0, *p* < 0.001, ε^2^ = 0.170), and loss of appetite (U = 263.0, *p* < 0.001, ε^2^ = 0.181) were each significantly associated with elevated nutritional risk, with patients reporting each symptom showing roughly double the mean risk score of those without it (Table 5). Heartburn showed no significant association (U = 155.0, *p* = 0.680, ε^2^ = 0.002), though this symptom was reported by only three patients, limiting statistical power for this comparison.

### 3.5. Nutritional Risk by Comorbidity

Chronic disease treatment was common: 74.5% of patients (*n* = 70) took regular medication, 28.7% (*n* = 27) were receiving chemotherapy, and 24.5% (*n* = 23) were receiving radiotherapy. Among individual comorbidities examined, hypertension (U = 648.0, *p* = 0.001, ε^2^ = 0.111; no disease M = 0.97 ± 1.26 vs. disease present M = 1.97 ± 1.48), atherosclerosis (U = 233.0, *p* = 0.005, ε^2^ = 0.084; M = 1.18 ± 1.34 vs. M = 2.55 ± 1.51), and chronic obstructive pulmonary disease (U = 79.0, *p* = 0.045, ε^2^ = 0.044; M = 1.28 ± 1.41 vs. M = 2.75 ± 0.96) were each significantly associated with elevated nutritional risk. Ischaemic heart disease (*p* = 0.430), asthma (*p* = 0.269), diabetes (*p* = 0.991), hypothyroidism (*p* = 0.747), and hyperthyroidism (*p* = 0.782) showed no statistically significant association with nutritional risk in this sample, notwithstanding modest numerical differences in some cases.

### 3.6. Nutritional Risk and Malignancy Diagnosis

Slightly under half of the sample (45.7%, *n* = 43) had a malignancy diagnosis; colorectal cancer was the most frequent single diagnosis (10.6%, *n* = 10), followed by gastric (6.4%), thyroid (5.3%), rectal (4.3%), breast (4.3%), and hepatic (4.3%) cancers, with smaller proportions of skin, small intestinal, oesophageal, and pancreatic cancer (Table 6). The proportion meeting the NRS 2002 threshold for nutritional treatment was higher among patients with malignancy (32.6%, 14/43) than among those without malignancy (15.7%, 8/51). The corresponding unadjusted risk ratio was 2.08 (95% CI: 0.96–4.47), but the association did not reach the prespecified significance threshold (Pearson χ^2^(1) = 3.70, *p* = 0.054, Cramér’s V = 0.199; Table 7). This observed numerical difference is uncertain and should be confirmed in a larger sample; it should not be interpreted as evidence of an independent association.

## 4. Discussion

### 4.1. Principal Findings

In this sample of 94 patients hospitalized in preparation for surgical treatment, roughly three-quarters showed no increased nutritional risk by NRS 2002, while approximately one in four met the tool’s threshold for nutritional treatment. In exploratory unadjusted analyses, higher scores were observed among older, widowed, retired, and less-educated patients, among those reporting nausea, vomiting, diarrhoea, or appetite loss, and among patients with hypertension, atherosclerosis, or chronic obstructive pulmonary disease. The proportion meeting the nutritional-treatment threshold was numerically higher among patients with malignancy, but the difference was not statistically significant (*p* = 0.054).

### 4.2. Comparison with Prior Studies

The proportion screening positive for nutritional risk in the present study (23.4%) sits toward the lower end of the wide 30–90% range reported by Szczygieł across chronically ill and oncological inpatients generally, though direct comparison is limited by differing screening instruments and patient populations across studies [4]. The present study also found an association between the nutritional risk score and the duration of ward stay recorded at questionnaire completion (H(3) = 36.18, *p* < 0.001). Because both the score and stay duration were examined cross-sectionally, the study cannot establish whether nutritional risk contributed to a longer stay, whether a longer stay was a marker of greater illness severity and higher nutritional risk, or whether both reflected another underlying factor.

Tojek and colleagues, applying NRS 2002 in a general surgery department, similarly found that patients at increased nutritional risk were significantly older than those not at risk (median age 72 versus 59 years, *p* < 0.05) [11], closely paralleling the strong age–risk correlation observed here (ρ = 0.633, *p* < 0.001). Tojek’s group additionally reported a significant association between urgent admission and elevated risk (48% of urgently admitted patients at risk versus 24% of those admitted on a planned basis, *p* < 0.05) [22]; the present study did not replicate this specific association (*p* = 0.828), despite a numerically higher mean risk score among urgently admitted patients, possibly reflecting differences in ward case-mix, sample size, or the specific surgical subspecialties represented across the two studies. Sznajder and colleagues, assessing patients admitted to a clinical oncology ward using both NRS 2002 and SGA, reported that sex was not a significant determinant of nutritional status [23], a finding directly consistent with the present study’s own null result for sex (*p* = 0.806). Recent work in older adults undergoing major pancreatic and biliary surgery has further shown substantial overlap between preoperative malnutrition, frailty, and sarcopenia, indicating that an elevated NRS 2002 score in an older patient may coexist with broader vulnerability not captured by nutritional screening alone [24].

### 4.3. The Contribution of Malignancy Diagnosis

The higher proportion of patients meeting the nutritional-treatment threshold among those with malignancy (32.6% versus 15.7%) is clinically consistent with prior work identifying cancer, particularly gastrointestinal cancer, as an important driver of hospital malnutrition [4,11,25]. Nevertheless, the present association did not reach statistical significance (*p* = 0.054; unadjusted RR = 2.08, 95% CI: 0.96–4.47), and the estimate should therefore be interpreted as an uncertain numerical difference rather than confirmatory evidence. Colorectal cancer was the single most common malignancy in the sample, and gastrointestinal tumours collectively accounted for the majority of recorded cancer diagnoses, consistent with mechanisms linking digestive-tract disease with reduced intake, absorption, and appetite [25]. National multidisciplinary recommendations for adult oncology patients emphasise early nutritional assessment and intervention [26]. Recent validation work in cancer surgery also confirms the clinical value of structured preoperative screening while showing that screening instruments vary in diagnostic performance across settings [27]. Poor preoperative nutritional status assessed using NRS 2002 and PG-SGA has been associated with postoperative infectious complications in gastric and colorectal cancer surgery [28], and ESPEN guidance recommends nutritional screening from diagnosis onward with escalation to assessment and intervention when abnormalities are identified [29].

### 4.4. Clinical and Educational Correlates

The observed unadjusted differences by educational level, marital status, and employment may reflect age or other social and clinical factors. However, age was not controlled for, and social support, food access, health literacy, and the ability to shop for or prepare meals were not measured. These mechanisms therefore remain hypotheses rather than explanations established by the present study. The practical importance of organizational and educational barriers is supported by recent nationwide data showing considerable variation between surgical departments in the implementation of perioperative nutritional screening and therapy for gastrointestinal cancer [30].

### 4.5. Strengths and Limitations

This study’s principal strength is its consistent use of NRS 2002 across a clinically diverse surgical population and its application of rank-based analyses appropriate to the observed score distributions. Its limitations include the single-centre setting, convenience sampling, the absence of a recorded refusal rate, the modest overall sample size, and small cell sizes in several comparison groups, including unemployed patients (*n* = 4), patients reporting heartburn, and patients hospitalized for more than 14 days (*n* = 2). These features reduce precision and generalisability. The malignancy result was not statistically significant (*p* = 0.054) and remains uncertain despite the observed numerical difference. Multiple bivariate comparisons were conducted without formal multiplicity correction, increasing the possibility of type I error; reported associations should therefore be regarded as exploratory and hypothesis-generating. Potential confounding by age, disease severity, and oncological case-mix was not addressed in a multivariable model because the study records did not support a stable adjusted analysis. The type and complexity of the planned surgical procedure were not collected, limiting clinical stratification of the heterogeneous sample. The cross-sectional assessment of ward-stay duration also precludes causal interpretation. Finally, NRS 2002 is a screening rather than diagnostic instrument; positive results should lead to comprehensive nutritional assessment, evaluation of muscle mass or frailty where appropriate, and an individualized perioperative nutrition plan [3,8,17,24].

## 5. Conclusions

In this cross-sectional study of 94 patients prepared for surgical treatment, most showed no increased nutritional risk by NRS 2002, but nearly one in four met the tool’s threshold for nutritional treatment. Exploratory unadjusted analyses showed higher nutritional risk scores among older patients and among some sociodemographic and clinical subgroups, but these associations may be confounded by age, disease severity, and other unmeasured factors. Patients with malignancy more frequently met the treatment threshold than those without malignancy, but the difference was not statistically significant and requires confirmation in a larger cohort. The cross-sectional association with duration of ward stay should not be interpreted causally. The findings support systematic preoperative NRS 2002 screening followed by comprehensive nutritional assessment of patients who screen positive.

## Figures and Tables

**Table 1:** Sociodemographic and baseline characteristics of 94 patients prepared for surgical treatment. Values are numbers (*n*) and percentages (%).

Characteristic	Category	*n*	%
Sex	Male	48	51.1
	Female	46	48.9
Age (years)	18–29	8	8.5
	30–40	6	6.4
	41–50	8	8.5
	51–60	15	16.0
	61–70	17	18.1
	71–80	30	31.9
	>80	10	10.6
Education	Primary	8	8.5
	Vocational	36	38.3
	Secondary	27	28.7
	Higher	23	24.5
Residence	Urban	47	50.0
	Rural	47	50.0
Marital status	Single	18	19.1
	Married	52	55.3
	Divorced	7	7.4
	Widowed	17	18.1
Employment	Employed	43	45.7
	Unemployed	4	4.3
	Retired	47	50.0
Living arrangement	Alone	20	21.3
	With spouse/children	52	55.3
	With other family	22	23.4
Regular medication	Yes	70	74.5
Chemotherapy	Yes	27	28.7
Radiotherapy	Yes	23	24.5
Malignancy	Present	43	45.7

Note: *n* = number of patients; % = percentage.

**Table 2:** Reasons for hospital admission among 94 patients prepared for surgical treatment. Values are numbers (N) and percentages (%).

Reason for Admission	N	%
Gastrointestinal disorder	53	56.4
Thyroid disorder	9	9.6
Abdominal/inguinal hernia	8	8.5
Appendectomy	6	6.4
Cholecystectomy	6	6.4
Breast tumour excision/mastectomy	4	4.3
Lower-limb varicose veins	3	3.2
Metastatic lymph node removal	3	3.2
Skin lesion excision	2	2.1

Note: N = number of patients; % = percentage.

**Table 3:** Final NRS 2002 nutritional risk classification among 94 patients prepared for surgical treatment. Values are numbers (N) and percentages (%).

NRS 2002 Classification	N	%
No increased nutritional risk	72	76.6
Nutritional treatment indicated (≥3 points)	22	23.4

Note: N = number of patients; % = percentage; NRS 2002 = Nutritional Risk Screening 2002.

**Table 4:** Unadjusted associations between sociodemographic and admission variables and the NRS 2002 total score among 94 patients prepared for surgical treatment.

Variable	Test	Statistic	*p*	Effect Size	Interpretation
Sex	Mann–Whitney U	U = 1071.0	0.806	ε^2^ = 0.010	Not significant
Place of residence	Mann–Whitney U	U = 915.0	0.152	ε^2^ = 0.011	Not significant
Education	Kruskal–Wallis	H(3) = 19.29	<0.001	ε^2^ = 0.181	Highest: primary
Marital status	Kruskal–Wallis	H(3) = 17.70	0.001	ε^2^ = 0.163	Highest: widowed
Employment	Kruskal–Wallis	H(2) = 21.92	<0.001	ε^2^ = 0.219	Highest: retired
Living arrangement	Kruskal–Wallis	H(2) = 4.50	0.106	ε^2^ = 0.027	Not significant
Age	Spearman correlation	ρ = 0.633	<0.001	—	Positive association
Admission mode	Mann–Whitney U	U = 1018.0	0.828	ε^2^ = 0.001	Not significant
Illness duration	Spearman correlation	ρ = 0.079	0.452	—	Not significant
Ward length of stay	Kruskal–Wallis	H(3) = 36.18	<0.001	ε^2^ = 0.369	Highest: 8–14 days

Note: H = Kruskal–Wallis statistic; U = Mann–Whitney statistic; *p* = *p*-value; ρ = Spearman rank correlation coefficient; ε^2^ = epsilon-squared effect size; NRS 2002 = Nutritional Risk Screening 2002; — = not reported or not applicable.

**Table 5:** Unadjusted associations between gastrointestinal symptoms or comorbidities and the NRS 2002 total score among 94 patients prepared for surgical treatment.

Variable	Test	Statistic	*p*	Effect Size	Interpretation
Diarrhoea	Mann–Whitney U	U = 315.0	0.002	ε^2^ = 0.100	Higher with symptom
Nausea	Mann–Whitney U	U = 341.5	<0.001	ε^2^ = 0.182	Higher with symptom
Vomiting	Mann–Whitney U	U = 316.0	<0.001	ε^2^ = 0.170	Higher with symptom
Loss of appetite	Mann–Whitney U	U = 263.0	<0.001	ε^2^ = 0.181	Higher with symptom
Heartburn	Mann–Whitney U	U = 155.0	0.680	ε^2^ = 0.002	Not significant
Hypertension	Mann–Whitney U	U = 648.0	0.001	ε^2^ = 0.111	Higher with disease
Atherosclerosis	Mann–Whitney U	U = 233.0	0.005	ε^2^ = 0.084	Higher with disease
COPD	Mann–Whitney U	U = 79.0	0.045	ε^2^ = 0.044	Higher with disease
Ischaemic heart disease	Mann–Whitney U	—	0.430	—	Not significant
Asthma	Mann–Whitney U	—	0.269	—	Not significant
Diabetes	Mann–Whitney U	—	0.991	—	Not significant
Hypothyroidism	Mann–Whitney U	—	0.747	—	Not significant
Hyperthyroidism	Mann–Whitney U	—	0.782	—	Not significant

Note: U = Mann–Whitney statistic; *p* = *p*-value; ε^2^ = epsilon-squared effect size; COPD = chronic obstructive pulmonary disease; NRS 2002 = Nutritional Risk Screening 2002; — = not reported or not applicable.

**Table 6:** Malignancy diagnoses reported among the 94 patients prepared for surgical treatment. Values are numbers (N) and percentages (%).

Cancer Type	N	%
None	51	54.3
Colorectal	10	10.6
Gastric	6	6.4
Thyroid	5	5.3
Rectal	4	4.3
Breast	4	4.3
Hepatic	4	4.3
Skin	3	3.2
Small intestine	3	3.2
Oesophageal	3	3.2
Pancreatic	1	1.1

Note: N = number of patients; % = percentage.

**Table 7:** Final NRS 2002 risk classification according to malignancy diagnosis status among 94 patients prepared for surgical treatment; the unadjusted risk ratio for nutritional-treatment indication was 2.08 (95% CI: 0.96–4.47).

Malignancy Status	No Risk N (%)	Treatment N (%)	Total
Cancer diagnosis present	29 (67.4)	14 (32.6)	43
No cancer diagnosis	43 (84.3)	8 (15.7)	51

Note: N = number of patients; % = percentage; NRS 2002 = Nutritional Risk Screening 2002; RR = unadjusted risk ratio; CI = confidence interval; χ^2^ = Pearson chi-square statistic; V = Cramér’s V.

## Data Availability

The data presented in this study are available on request from the corresponding author.
